# A Concise View of All the Most Important Facts Which Have Hitherto Appeared Concerning the Cow-Pox

**Published:** 1801-04

**Authors:** 


*A Concise View of all the most Important Facts which have hitherto appeared concerning the Cow-pox.* By C. R. AIKIN, Member of the Royal College of Surgeons in London. 12mo. pp. 102, price 2s. 6d. London, 1801, R. Phillips, &c.

THIS practical Manual appears to us to be admirably calculated to anfwer the wifhes of Country Practitioners, and Surgeons in the Navy and Army; and as the fale is very extenfive, and new editions are intended to be given frequently, in order that the lateft and moft accurate information may at all times be found in it; we take the liberty of fubmitting the following Hints for the next edition.

1ft. A new plate.

Page 19. On the Origin of the Cow-pox as derived from the Horfe, Mr. A. has not mentioned the fimilarity of the equine and vaccine puftule, nor the fecurity the conftitution receives from the inoculation of equine fluid.

Page 23, line 13, for, *is not* rendered thereby, &c. read, *has been* rendered, &c.

Page 38, line 10, formation of *matter*, read, formation of a *vesicle*, or let the term *matter* be explained.

Page

Page 39, line 5, "rendered much milder by *inoculation.*" The Cow-pox cannot be communicated by any other means than thofe of *inoculation*, and therefore no comparifon between the Small-pox and Cow-pox can be admitted on that ground. See p. 44, 45.

Page 57, line 9, and page 58, line 5, &c. This advice is too general, and requires to be more accurately and pointedly circumfcribed.

Page 66, line 8. The conftitution is moft commonly affected on the fourth and fifth days.

Page 75, line 10, &c. and note, page 76. There are no puftular cafes refembling Small-pox, derived from real Vaccine fluid.

Page 99, line 17. "Dr. Jenner's able *investigation.*" It is but an act of juftice due to the public and Dr. Jenner, that every writer on this fubject, efpecially in this country, fhould declare unequivocally and explicitly, that Dr. Jenner is entitled to the admiration and reward of a grateful public, not merely for inveftigating the nature of a known difeafe, but for conceiving and perfecting the important idea of employing that difeafe, divefted of its feverity by art, to fuperfede and finally annihilate the Small-pox. All foreigners have hitherto done him this juftice.

